# Leptin Prevents Lipopolysaccharide-Induced Depressive-Like Behaviors in Mice: Involvement of Dopamine Receptors

**DOI:** 10.3389/fpsyt.2019.00125

**Published:** 2019-03-12

**Authors:** Rafaela Carneiro Cordeiro, Adriano José Maia Chaves Filho, Nayana Soares Gomes, Viviane de Sousa Tomaz, Camila Dantas Medeiros, Ana Isabelle de Góis Queiroz, Michael Maes, Danielle S. Macedo, Andre F. Carvalho

**Affiliations:** ^1^Neuropharmacology Laboratory, Department of Physiology and Pharmacology, Universidade Federal do Ceará Fortaleza, Brazil; ^2^McGill Group for Suicide Studies, Douglas Mental Health Institute, McGill University Montreal, QC, Canada; ^3^Department of Psychiatry, Faculty of Medicine, Chulalongkorn University Bangkok, Thailand; ^4^Department of Psychiatry, University of Toronto Toronto, ON, Canada; ^5^Centre for Addiction and Mental Health Toronto, ON, Canada

**Keywords:** leptin, depression, LPS, dopamine, prefrontal cortex, psychiatry

## Abstract

Depression is a chronic and recurrent disorder, associated with high morbidity and risk of suicide. Leptin was firstly described as an anti-obesity hormone, but several actions of leptin in CNS have been reported. In fact, leptin regulates dopaminergic neurotransmission in mesolimbic areas and has antidepressant-like properties in stress-based models. In the present study, we investigated, for the first time, putative antidepressant-like effects of leptin in an animal model of depressive-like behaviors induced by lipopolysaccharide (LPS), and the potential involvement of dopamine receptors as mediators of those behavioral effects. Mice were injected leptin (1.5 mg/kg, IP) or imipramine prior to LPS administration. To evaluate the involvement of dopamine receptors, different experimental groups were pretreated with either the dopaminergic antagonist SCH23390, for D1 receptors or raclopride, for D2/D3 receptors, prior to leptin injection. Twenty-four hours post-LPS, mice were submitted to the forced swimming and sucrose preference tests. In addition, IL-1β levels were determined in the prefrontal cortex (PFC), hippocampus and striatum. BDNF levels were measured in the hippocampus. Our results showed that leptin, similarly to imipramine, prevented the core behavioral alterations induced by LPS (despair-like behavior and anhedonia), without altering locomotion. In neurochemical analysis, leptin restored LPS-induced changes in IL-1β levels in the PFC and striatum, and increased BDNF levels in the hippocampus. The blockade of dopamine D1 and D2/D3 receptors inhibited leptin's antidepressant-like effects, whilst only the blockade of D1-like receptors blunted leptin-induced increments in prefrontal IL-1β levels. Our results indicate that leptin has antidepressant-like effects in an inflammatory model of depression with the contribution, at least partial, of dopamine receptors.

## Introduction

Depression is the third leading source of years lived with disability worldwide and has a lifetime prevalence of 14.6% among people living in high-income countries (1, 2). In spite of the substantial burden associated with depression, only approximately a third of patients achieve remission after an adequate trial with first-line monoaminergic antidepressants (3). Therefore, the search of potential antidepressant compounds with genuinely novel mechanisms of action is an unmet need in the field (4).

Accumulating evidence indicates that peripheral immune activation and neuroinflammation may contribute to the development of depression (5, 6). For example, a recent meta-analysis evidenced that individuals with depression exhibit elevated peripheral levels interleukin (IL)-6, tumor necrosis factor (TNF)- α, IL-10, the soluble IL-2 receptor, C-C chemokine ligand 2, IL-13, IL-18, IL-12, the IL-1 receptor antagonist, and the soluble TNF receptor 2 compared to healthy controls (7). Furthermore, treatment resistant depression was accompanied by high inflammation (C-reactive protein >3 mg/l) in almost half of the patients enrolled in a previous study (8). Conversely, antidepressants may mitigate peripheral inflammation although it remains unclear whether anti-inflammatory mechanisms may predict antidepressant response (9). Peripheral immune activation and inflammation may contribute to the emergence of depression through several mechanisms including the activation of the kynurenine pathway (10), alterations in neuronal plasticity (11), microglial activation (12), and neuroendocrine effects (13).

Several lines of evidence also point to the involvement of Gram-negative bacteria in the pathophysiology of depression. For example, depression accompanied by high plasma IgA responses to antigens/lipopolysaccharide (LPS) of commensal gut bacteria and increased root canal LPS in patients with comorbid depression and chronic apical periodontitis (14, 15). Moreover, increased translocation of Gram-negative bacteria in depression is associated with higher levels of immune activation and nitro-oxidative stress (16, 17). The systemic administration of a Gram-negative bacterial lipopolysaccharide (LPS) also leads to microglial activation and subsequent increments in production of cytokines (18, 19).

Indeed, the animal model of depressive-like behaviors induced by the systemic administration of LPS was first proposed by Yirmiya (20). Earlier studies also showed that LPS administration may induce activation of various pathways associated with depression (21, 22). In humans and rodents, a unique systemic administration of LPS causes a spectrum of behavioral alterations that resembles depression, including depressed mood, fatigue, and psychomotor slowing in humans (23, 24), as well as anhedonia, decreased activity, cognitive dysfunction, and altered sleep in rodents (25, 26). Also, LPS-induced model has been increasingly used for the screening of new antidepressants because it induces important neurochemical and structural alterations in brain areas related to mood regulation (22, 27).

Abnormalities in the reward brain circuit have been well-described in depressive patients as well as in several animal models of depression (28, 29). Depressed individuals generally rate stimuli of positive valence as being less positive, less arousing, or less able to affect their mood (30). Neurobiological studies on anhedonia have focused primarily in neural substrates involved in motivation and reinforcement (“wanting”). In this regard, the neurotransmitter dopamine in brain sub-cortical structures, particularly the nucleus accumbens (NAcc), ventral tegmental area (VTA), amygdala and hippocampus, as well as in cortical regions such as the ventromedial prefrontal cortex (vmPFC) and orbital frontal cortex regulates motivation and reinforcement (31).

Neuroinflammation triggers alterations in dopamine brain circuits. In this regard, depressive behavior following interferon (IFN)-α administration to rhesus monkeys was related to lower cerebrospinal fluid (CSF) concentrations of the dopamine metabolites, homovanillic acid (HVA) and 3,4-dihydroxyphenylacetic acid (DOPAC) (32). Indeed, a number of experimental studies have pointed toward a decrease in striatal dopamine release (including from NAcc) in animals challenged with inflammatory cytokines, such as IL-1 β and IFN-α which correlates with decreased sucrose preference (impaired motivation) (33, 34).

Besides regulating motivated behavior, consistent evidence suggests that mesolimbic dopaminergic system also influences important aspects of feeding behavior and energy expenditure (35). Indeed, appetite and metabolic changes associated with depression range from severe hypophagia and anorexia to binge eating and obesity. In this context, the hormone leptin has received great attention (36). Leptin was initially described as an anti-obesity hormone, acting through a negative feedback loop between adipose tissue and the hypothalamus to control energy homeostasis (37). Beyond hypothalamus, the long form of the leptin receptor (ObRb) is expressed in several brain areas, such as the NAcc, VTA, hippocampus and prefrontal cortex (PFC) (38, 39). Interestingly, leptin regulates dopaminergic neurotransmission in these limbic structures by controlling firing rates of dopaminergic neurons in VTA as well as by regulating feeding behavior via dopamine D2 receptors (40).

The role of leptin in depression has been extensively investigated. Some studies have demonstrated that chronic stress paradigms impair serum leptin levels, and the deletion of its receptor (long form) in hippocampus and VTA induces depressive- and anxiety-like behaviors (41, 42). Also, the direct antidepressant properties of leptin have been demonstrated both by peripheral as well as by in intrahippocampal injection (43, 44). These behavioral effects of leptin were accompanied by increased neurogenesis in the hippocampus and reversion of corticoid-induced alterations in intracellular signaling pathways, such as GSK3β/β-catenin (44). However, a recent meta-analysis showed that peripheral levels of leptin did not significantly differ between patients with depression and healthy controls although heterogeneity was large and body mass index of participants across studies emerged as a significant moderator (45).

Despite the well-known effects of leptin on dopamine signaling, to our knowledge, the involvement of dopamine receptors on leptin-induced antidepressant-like effects remain unexplored. Therefore, based on the putative role of dopaminergic signaling in the central effects of leptin and the absence of studies exploring the effects of this hormone in animal model of depressive-like behavior induced by immune challenge, we decided to test the hypothesis that leptin could prevent LPS-induced depressive-like behavior in mice and that this effect is mediated, at least partly, by the participation of D1- and/or D2-like dopamine receptors. Furthermore, we assessed whether leptin's antidepressant-like effects could be associated with alterations in IL-1β levels in limbic brain areas (PFC, hippocampus and striatum) and/or hippocampal BDNF levels in the brain of mice challenged with LPS.

## Materials and Methods

### Animals

The experiments were performed in a total of 216 male Swiss mice (20–30 g) obtained from the Animal House of the Universidade Federal do Ceará. Animals were housed 8 per cage under standard animal housing conditions, polycarbonate cages (42 × 20.5 × 20 cm), temperature 22 ± 1°C, humidity 60 ± 5%, 12 h light-dark cycle (light on 6 a.m.) and food/water *ad libitum*. All experimental procedures were performed between 8 a.m. and 2 p.m., according to the Guide for the Care and Use of Laboratory Animals, from the US Department of Health and Human Services (46) and to the Brazilian legislation on animal experimentation (law n° 11.794). This research protocol was approved by the local ethic committee of Universidade Federal do Ceará. All efforts were made to minimize animal suffering and to reduce the number of animals used.

### Drugs

Lipopolysaccharide (LPS) from *Escherichia coli*, strain 055:B5 and R(+)-SCH23390 hydrochloride were obtained from Sigma-Aldrich Corp., St. Louis, USA. Recombinant Mouse Leptin Protein was purchased from R & D Systems. Raclopride was obtained from Tocris Bioscience, Bristol, United Kingdom. The drugs were administered at the following doses (mg/kg): LPS 0.5, leptin 1.5, imipramine 10, SCH23390 0.015 and raclopride 0.04. All drugs were freshly prepared and diluted in saline solution. All other chemicals used were of analytical grade.

### Experimental Design

**Figure 1:**
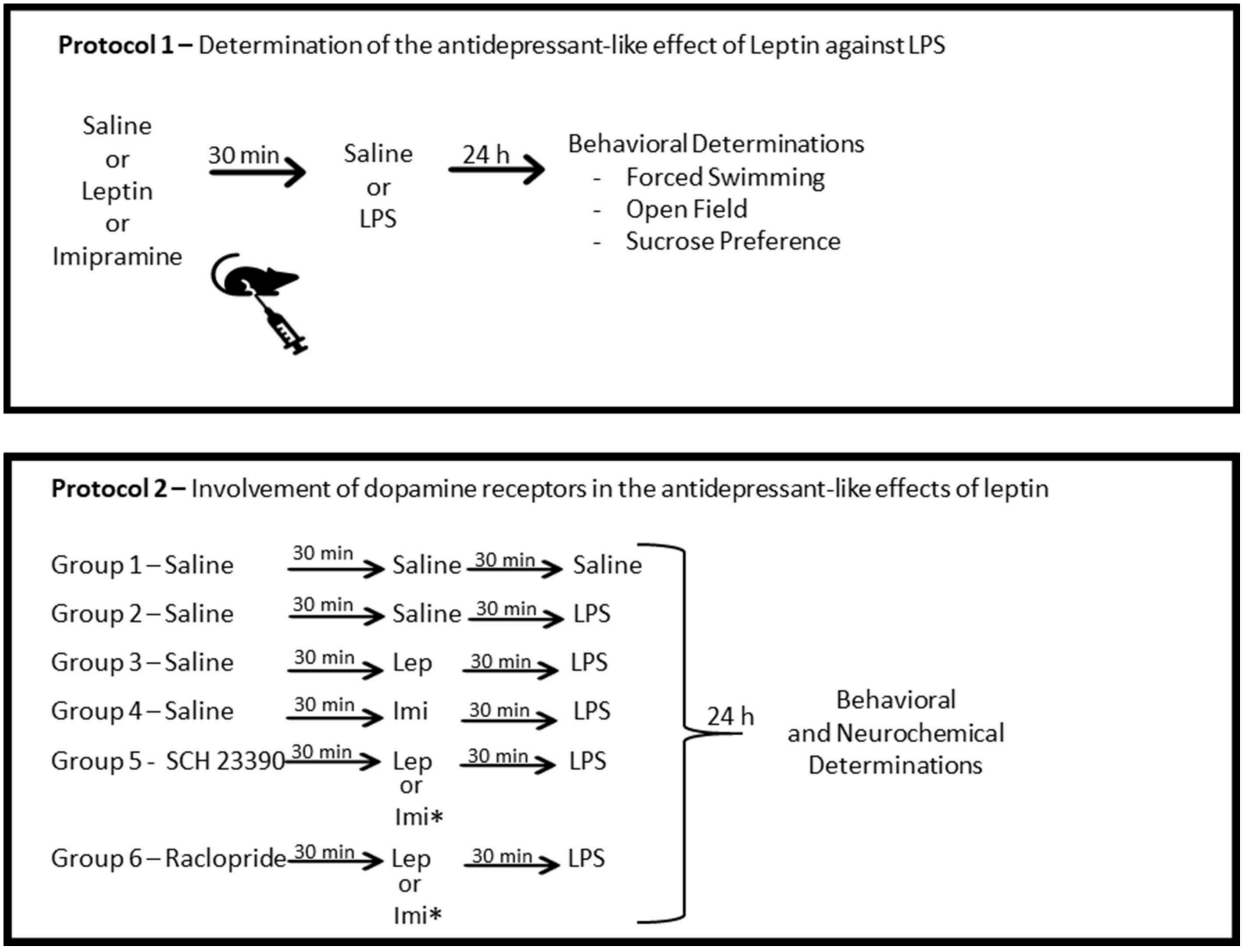
Schematic representation of the experimental design. Protocol 1 was designed to determine the antidepressant-like effects of leptin against LPS-induced depressive-like behavior. Protocol 2 was designed to determine the participation of D1-like dopamine receptors, by the pre-administration of the antagonist SCH23390 and of D2/D3 dopamine receptors by the administration of the antagonist, raclopride. ^*^This group was submitted only to behavioral determinations. Lep, leptin; Imi, imipramine.

The first protocol of the study consisted of behavioral determinations to test the antidepressant-like effect of leptin in LPS acute model (Figure 1). To do this, mice were randomly divided into six groups of 20 animals each. The study comprised the following groups submitted to intraperitoneal (IP) administration at the volume of 0.1 ml/10 g body weight, as follows: saline (control) group—received two doses of saline; leptin group—received leptin (1.5 mg/kg) prior to saline; imipramine group—received imipramine (10 mg/kg) prior to saline; LPS group—received saline prior to LPS (0.5 mg/kg); Lep ± LPS group—received leptin prior to LPS; Imi ± LPS group—received imipramine prior to LPS. In all situations the administration of the drugs was separated by a 30 min interval. The behavioral determinations were performed 24 h after the last administration of saline or LPS. LPS dose (0.5 mg/kg) was chosen based on previous studies evaluating depressive-like behavioral changes and neurochemical alterations in mice (22, 25). The dose of leptin (1.5 mg/kg) was selected based on preclinical studies evaluating the antidepressant-like activity of this hormone (43, 44). Imipramine was used as standard antidepressant (25, 27).

The second protocol was designed to determine the involvement of dopamine receptors in the antidepressant-like effects of leptin, by a systemic blockage of D1-like or D2-D3 receptors (Figure 1). In this protocol, the animals were divided into groups of 24 animals/group treated as follows: saline (control) group—received three injections of saline; LPS group—received two injections of saline prior to the administration of LPS; Lep ± LPS group—received one injection of saline followed by leptin and LPS; SCH ± Lep ± LPS group—received one injection of SCH followed by leptin and LPS; Rac ± Lep ± LPS—received raclopride prior to leptin and LPS; Imi ± LPS group—received saline prior to imipramine and LPS; SCH ± Imi ± LPS group—received one injection of SCH followed by imipramine and LPS; Rac ± Imi ± LPS group—received raclopride prior to imipramine and LPS. In all situations, a 30 min interval between injections was used. The doses of the antagonists, SCH23390 (0.015 mg/kg, IP, D1-like receptor antagonist) and raclopride (Rac−0.04 mg/kg, IP, D2-D3 receptor antagonist), were chosen based on previous studies (35, 47). Again, 24 h after the last administration of saline or LPS, animals were subjected to the behavioral tests.

Open field, forced swimming or sucrose preference tests were conducted to assess behavioral alterations. For these tests the animals were assigned into two distinct sets. The first set of mice comprised 6–10 animals/group, which were tested in the open field and forced swimming tests, in this order. The second set of animals was used for sucrose preference test comprising 6 animals/group. Distinct groups of animals were treated with the drugs and sacrificed for neurochemical determinations. The brain areas prefrontal cortex (PFC), hippocampus and striatum were dissected and immediately stored at −80°C until assayed. As hippocampus is one the main area that maintain active neurogenesis in adult brain, a mechanism involved in the response to antidepressants (48), we decided to evaluate BDNF levels in this area.

The primary outcome of this study was to determine changes in locomotion, behavioral despair (by the forced swimming test) and anhedonia (by the sucrose preference test) in animals administered leptin as well as the participation of dopamine receptors. The secondary outcome was to verify alterations in inflammatory cytokines and BDNF in brain areas related to mood regulation.

### Behavioral Tests

#### Forced Swimming Test

In this test, animals were subjected individually to the analysis of the depressive-like behavior, based on a model adapted from Porsolt et al. (49). In this task the immobility period of the animal (during 5 min) is registered after a 1-min period of habituation and, the greater this time, the lower the animal's motivation to escape, representing, thus, a depressive-like behavior (50). Animals were placed individually inside an acrylic cylinder (25 cm high, 10 cm diameter and 8 cm depth) containing water at 25°C. Mice were unable to escape or touch the bottom of the cylinder. Any mouse appearing to have difficulty keeping its head above the water was removed from the cylinder and excluded from the study.

#### Open Field Test

This test was adapted from the model initially proposed by Archer (51) and was used here to evaluate the animals' locomotor activity and anxiety-like behavior (52). The experimental trial was conducted in a dark room with red light where the mice were individually placed in a transparent acrylic box (80 × 80 × 30 cm) divided equally into 9 quadrants. Each animal was placed in the center of the arena immediately before the test and allowed to explore it for 5 min. The number of squares crossed by the animals was registered and used as a parameter of locomotor activity whereas the time spent in the center of the field was used for the determination of anxiety-like behavior. The arena was cleaned with a 5% ethanol solution between each test animal. The experiment was videotaped and analyzed by two raters blinded to the treatment groups.

#### Sucrose Preference Test

The test was performed 24 h after LPS administration to evaluate anhedonia. The procedure consisted of an adaptation period 72 h before the test in which mice were trained to adapt to sucrose solution with two bottles of 1% (w/v) sucrose solution placed in each cage. Twenty-four hours later, sucrose solution in one bottle was replaced with tap water during 24 h. After this adaptation period, mice were deprived of water and food for further 24 h. For the sucrose preference test mice were housed in individual cages with free access to two bottles containing 100 ml of sucrose solution (1% w/v) and 100 ml of water. After 1 h, the volumes of consumed sucrose solution and water were recorded and the sucrose preference was calculated as follows: % sucrose consumption = sucrose consumption/(water + sucrose consumption) × 100 (53).

### Neurochemical Determinations

#### Immunoassay for IL-1β

Each brain area was homogenized in 8 volumes of PBS buffer with protease (EMD Biosciences) and phosphatase (Sigma-Aldrich) inhibitors and centrifuged (10,000 rpm, 5 min). For IL-1β determinations 50 μL samples were used. The immunoenzymatic assay (ELISA) was performed according to the manufacturer's protocol (R&D systems, Minneapolis, MN, USA) and expressed in pg/g tissue.

#### Immunoassay for BDNF

Each hippocampus was homogenized in 20 volumes of PBS buffer with protease (EMD Biosciences) and phosphatase (Sigma-Aldrich) inhibitors and centrifuged (10,000 rpm, 5 min). For BDNF determinations 50 μL samples were used. The immunoenzymatic assay (ELISA) was performed according to the manufacturer's protocol ELISA (Merck Millipore, Merck KGaA, Darmstadt, Germany) and expressed in pg/g tissue.

### Statistical Analyses

Data from behavioral and neurochemical determinations are present as mean ± S.E.M. (standard errors of the mean). Kolmogorov–Smirnov normality test was conducted to verify the normal distribution of data. Statistical analyses were conducted by regular one- or two-way ANOVA with Tukey's multiple comparisons as *post-hoc* test. In the case of two-way ANOVA, the factors “LPS model” (saline and LPS) and “drug treatment” (control, leptin and imipramine) were used. The significance level was set at *P* ≤ 0.05. GraphPad Prism 6.0 Version for Windows, GraphPad Software (San Diego, CA, USA) was used.

## Results

**Figure 2:**
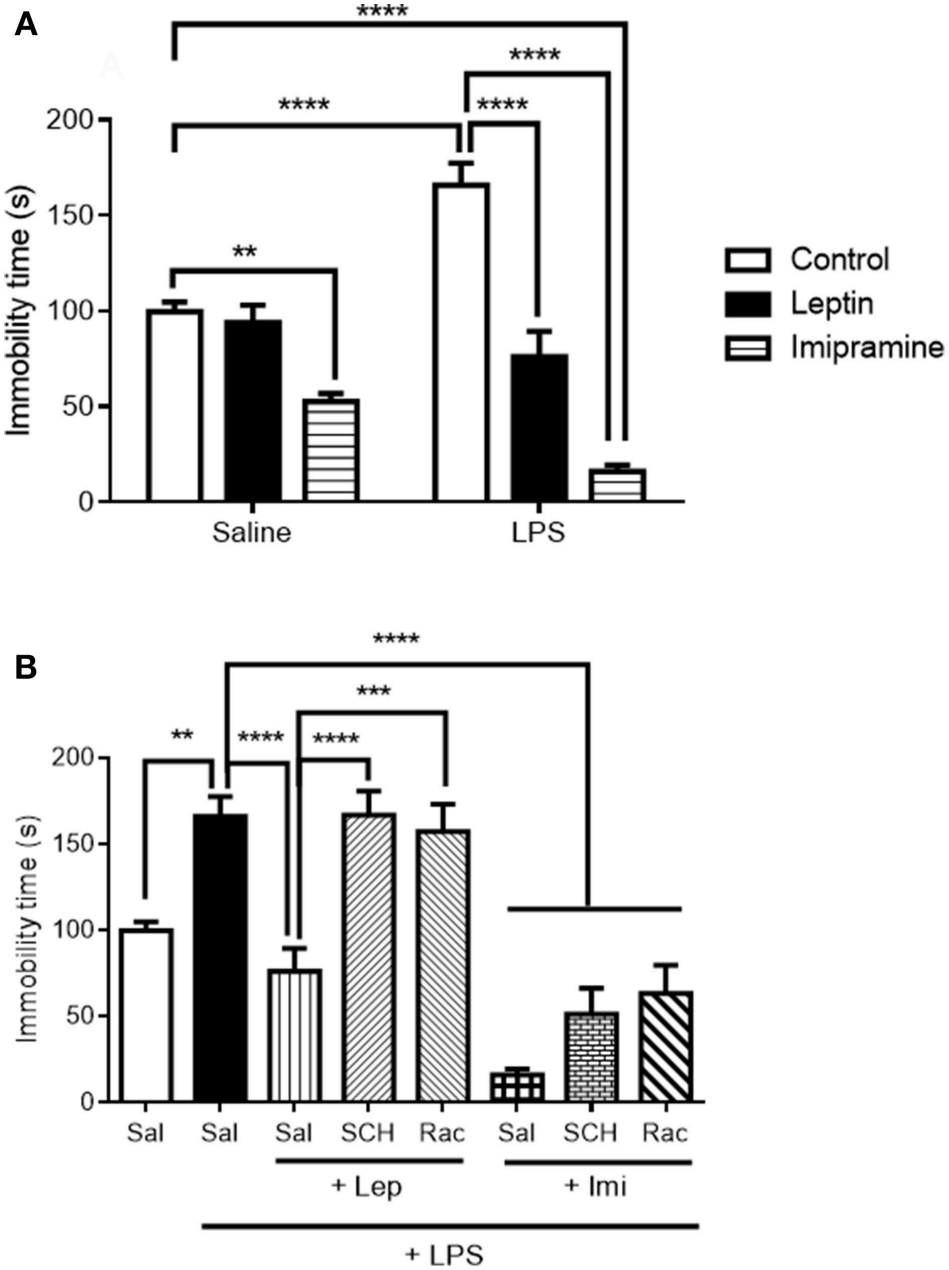
Immobility time (s) in the forced swimming test (FST) of animals submitted to protocol 1 **(A)** and protocol 2 **(B)**. Bars represent mean ± standard error of the mean (S.E.M.) of 6–10 animals/group. The results in part A were analyzed by two-way ANOVA, while in part B by one-way ANOVA, in both cases with Tukey's as *post-hoc* test. ^**^P < 0.01, ^***^P < 0.001, ^****^P < 0.0001 as indicated by the connectors. Sal, saline; SCH, SCH23390; Rac, raclopride; Lep, leptin, Imi, imipramine; LPS, Lipopolysaccharide.

As a first step, we evaluated the antidepressant-like effect of leptin and of the standard antidepressant imipramine in the forced swimming test (Figure 2A). Two-way ANOVA revealed a significant interaction between the factors “LPS model” and “drug treatment” [*F*_(2, 42)_ = 19.61, *P* < 0.0001], with significant main effect of “drug treatment” [*F*_(2, 42)_ = 60.66, *P* < 0.0001]. As expected, LPS alone significantly increased immobility time (*P* < 0.0001) when compared to control animals. Pretreatment with leptin kept immobility time akin to control animals (*P* < 0.0001 in relation to LPS-challenged group). On the other hand, imipramine decreased mice immobility time in relation to control group when administered alone (*P* = 0.0070) and when administered before LPS (Imi+LPS group) in relation to LPS-challenged group (*P* < 0.0001).

In the evaluation of forced swimming in animals submitted to the second protocol (Figure 2B), pre-administration of D1-like or D2/D3 receptor antagonists abrogated leptin antidepressant-like effects (SCH+Lep+LPS vs. Lep+LPS, *P* < 0.0001; Rac+Lep+LPS vs. Lep+LPS, *P* = 0.0002). On the other hand, neither D1-like nor D2/D3 receptor antagonists prevented the reduction in immobility time observed in Imi+LPS group in relation to LPS group [one-way ANOVA: (*F*_(7, 53)_ = 22.80, *P* < 0.0001)].

**Figure 3:**
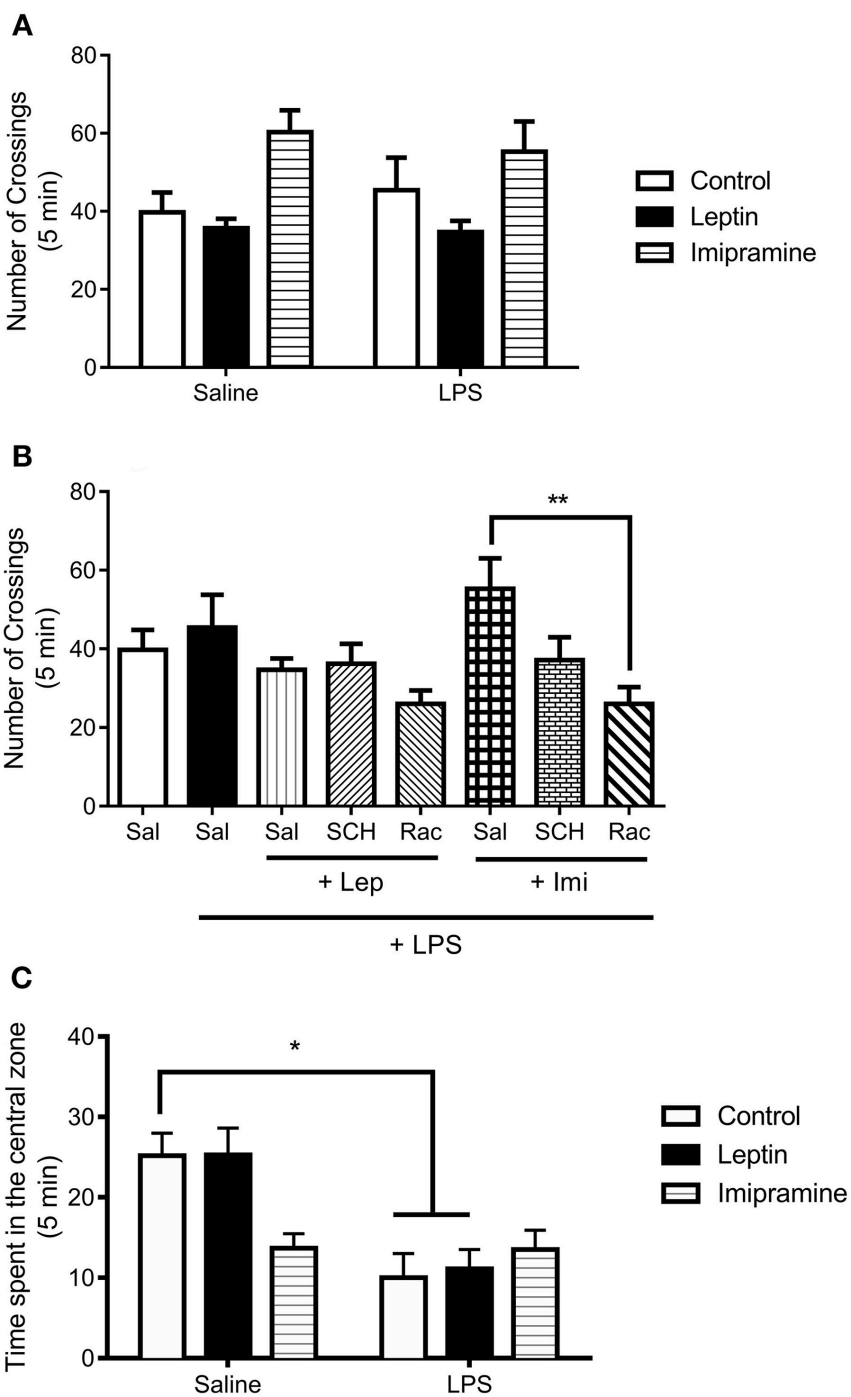
Number of crossings in the open field test of animals submitted to protocol 1 **(A)** and protocol 2 **(B)**. Time spent in the center of the open field **(C)**. Bars represent mean ± standard error of the mean (S.E.M.) of 6–10 animals/group. The results in part A and C were analyzed by two-way ANOVA, while in part B by one-way ANOVA, in both cases with Tukey's as *post-hoc* test. ^*^P<*0.05*, ^**^P < 0.01 as indicated by the connectors. Sal, saline; SCH, SCH23390; Rac, raclopride; Lep, leptin, Imi, imipramine; LPS, Lipopolysaccharide.

We next determined alterations in the locomotor activity to better address LPS-induced sickness behavior in these mice, a complex behavioral phenotype associated with hypolocomotion (Figure 3). In our results, we observed no significant interaction between the factors “LPS model” and “drug treatment” [*F*_(2, 37)_ = 0.4668, *P* = 0.6307] (Figure 3A). This result suggests that the animals used in the present study were not in sickness behavior. In the evaluation of the involvement of dopamine receptors in the locomotor activity of the animals (Figure 3B), we only observed a significant decrease in the number of crossings in the group Rac+Imi+LPS in relation to Imi+LPS (*P* = 0.0038). Still in the open field test, the measure of anxiety-like behavior evaluated by the time spent in the center of the field (Figure 3C) revealed a significant main effect of “drug treatment” [*F*_(1, 41)_ = 14.22, *P* = 0.0005]. We observed that LPS-treated and Lep+LPS mice presented a decrease in the time spent in the center of the field in relation to saline-treated mice (*P* < 0.05).

**Figure 4:**
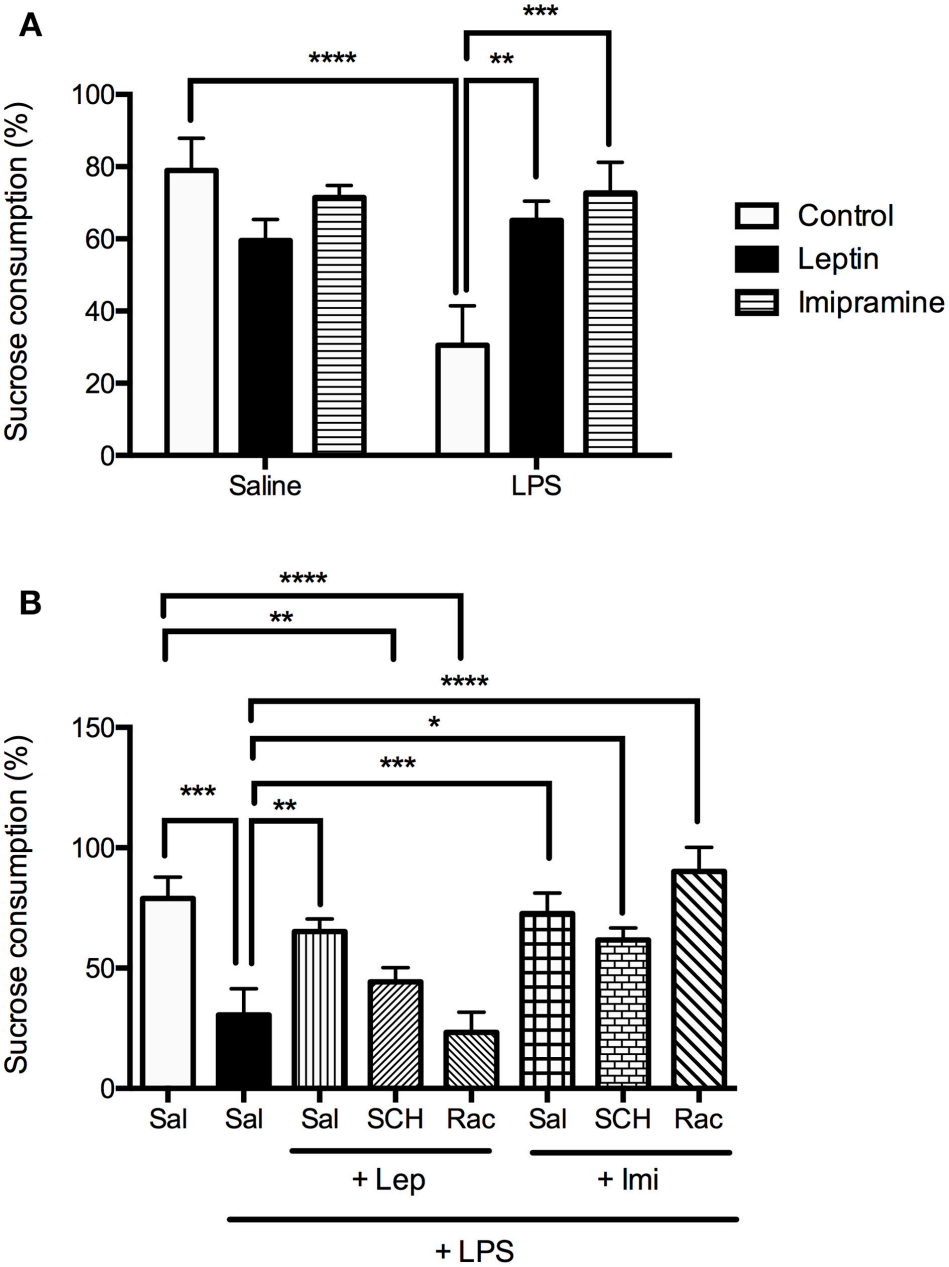
Sucrose preference measured as % of sucrose consumption of animals submitted to protocol 1 **(A)** and protocol 2 **(B)**. Bars represent mean ± standard error of the mean (S.E.M.) of 6 animals/group. The results in part A were analyzed by two-way ANOVA, while in part B by one-way ANOVA, in both cases with Tukey's as *post-hoc* test. ^*^P < 0.05, ^**^P < 0.01, ^***^P < 0.001, ^****^P < 0.0001 as indicated by the connectors. Sal, saline; SCH, SCH23390; Rac, raclopride; Lep, leptin, Imi, imipramine; LPS, Lipopolysaccharide.

To better evaluate LPS-induced anhedonia, sucrose preference test was performed (Figure 4). In Figure 4A we observed a significant interaction between the factors “LPS model” and “drug treatment” [*F*_(2, 29)_ = 7.729, *P* = 0.0020], with significant main effect of “LPS model” [*F*_(1, 29)_ = 4.883, *P* = 0.0352]. The results showed that 24 h after the endotoxin administration, mice displayed a significant decrease in sucrose preference when compared to control group (*P* < 0.0001). The administration of leptin (*P* = 0.0043) or imipramine (*P* = 0.0004) prevented the decrease in sucrose preference induced by LPS.

In the second protocol (Figure 4B), the percentage of sucrose consumption in the groups SCH+Lep+LPS (*P* = 0.0026) or Rac+Lep+LPS (*P* < 0.0001) was lower than the observed in the control group, being like LPS group, revealing that both D1-like and D2/D3 antagonism abrogate the effect of leptin in this test. Imipramine (*P* = 0.0004), as leptin (*P* = 0.0039), prevented the decrease in sucrose preference in LPS-treated animals, although pretreatment with D1 and D2/D3 receptor antagonists did not change the effects of imipramine in sucrose consumption [one-way ANOVA: (*F*_(7, 36)_ = 10.06, *P* < 0.0001)].

**Figure 5:**
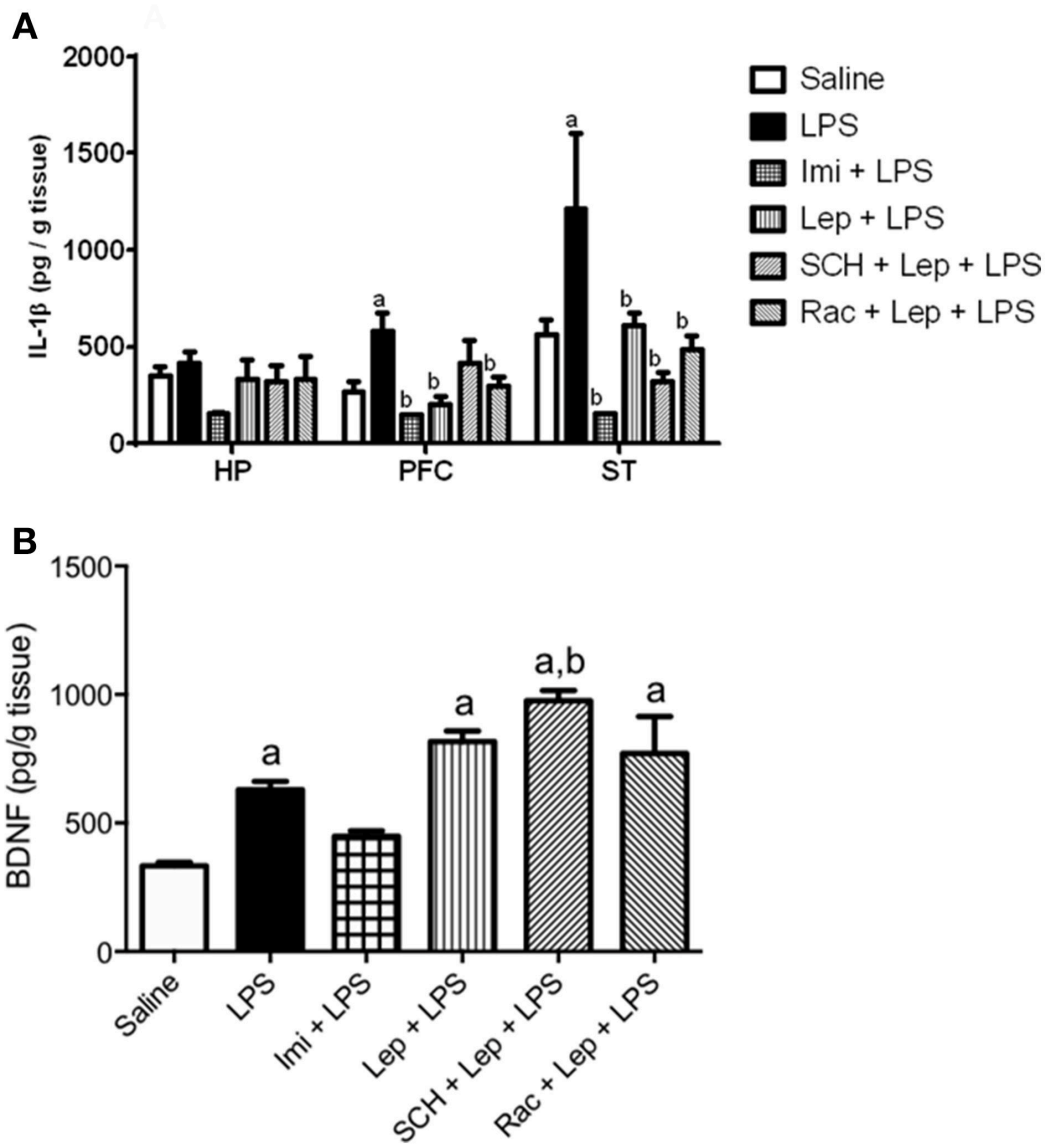
**(A)** Levels of IL-1β in the prefrontal cortex, hippocampus and striatum and **(B)** BDNF levels in the hippocampus of animals of animals submitted to protocol 2. Bars represent mean ± standard error of the mean (S.E.M.) of 6–7 animals/group. ^a^*P* < 0.05 vs. control, ^b^*P* < 0.05 vs. LPS according to one-way ANOVA followed by Tukey's multiple comparison test. SCH, SCH23390; Rac, raclopride; Lep, leptin; Imi, imipramine; LPS, Lipopolysaccharide; HP, hippocampus; PFC, prefrontal cortex; ST, striatum; IL-1β, interleukin−1β; BDNF, brain-derived neurotrophic factor.

Since LPS-induced depressive-like behavior is related to the induction of pro-inflammatory cytokines in the brain, the levels of IL-1β were measured (Figure 5A). We found that IL-1β levels were increased in LPS treated animals when compared to saline group in the PFC (*P* = 0.0287) [one-way ANOVA: (*F*_(5, 31)_ = 5.743, *P* = 0.0007)] and striatum (*P* = 0.038) [one-way ANOVA: (*F*_(5, 30)_ = 6.498, *P* = 0.0003)]. Pretreatment with imipramine (*P* < 0.0001) or leptin (*P* < 0.001) significantly decreased IL-1β levels in these brain areas when compared to LPS group. The antagonism of D1-like receptors partially inhibited the effects of leptin in PFC IL-1β levels. We did not observe a significant difference in this parameter in the group SCH+Lep+LPS in relation to Sal+LPS or control groups. No significant difference was observed in the evaluation of IL-1β levels in the hippocampus.

Finally, because oxidative stress and inflammation alters the brain levels of neurotrophic factors, BDNF content was evaluated in the hippocampus (Figure 5B), the main site of neurogenesis in the adult brain. Twenty-four hours post-LPS injection, BDNF levels were increased in LPS-treated group when compared to controls (*P* = 0.0178). The group Lep+LPS also presented increased levels of BDNF when compared to control (*P* = 0.0003), while in the group pretreated with imipramine there was no significant alteration. The groups treated with SCH+Lep+LPS (*P* < 0.0001) or Rac+Lep+LPS (*P* = 0.0005) maintained the increase in BDNF levels observed in Lep+LPS treated mice being the results significant in relation to saline group (*P* < 0.05). In the case of SCH+Lep+LPS group, the increase in BDNF levels was also significant in relation to Sal+LPS group (*P* = 0.0041) [one-way ANOVA: (*F*_(5, 37)_ = 19.99, *P* < 0.0001)].

## Discussion

In the present study, we are showing the antidepressant-like effects of leptin in LPS-induced inflammatory model of depression through two established behavioral paradigms, namely forced swimming and sucrose preference test. We also, demonstrated the involvement of D1-like and D2/D3 dopamine receptors in the behavioral effects of leptin, as well as in the ability of this hormone to rescue LPS-induced increases in IL-1β levels in the PFC and striatum. Interestingly, D1-like receptors blockade blunted the effects of leptin on IL-1β levels in the PFC, but not in the striatum. Additionally, hippocampal BDNF levels were increased in leptin-treated mice and unaffected by the blockade of dopamine receptors. Regarding anxiety-like behavior evaluated in the open field test, leptin was not able to prevent LPS-induced anxiety-like behavior. Therefore, this study brings new evidence about the antidepressant-like properties of leptin, while suggests the participation of dopaminergic receptors in the behavioral effects of this hormone as well as the participation of D1-like receptors in its anti-inflammatory effect in the PFC.

Leptin's antidepressant-like effect was already demonstrated in stress models of depression, such as chronic unpredictable stress and chronic social defeat. Indeed, this previous study showed that animals exposed to stress-induced depression present low plasma levels of leptin, while leptin treatment restores these parameters (43). These authors also found that the hippocampus was the main site of leptin antidepressant-like action (43). Based on the results obtained in our study we can infer that other areas of the limbic system, such as striatum and PFC also contribute to leptin antidepressant effect.

Regarding LPS-induced depressive-like behavior, it is well known that animals injected with LPS develop an acute mild state of nosothymia, termed sickness-behavior. However, 24 h post LPS, a state of depressive-like behavior emerges, a time-point when motor activity and food and drink consumption return to normal (6, 22). Accordingly, in our results, the animals challenged with LPS and pretreated with leptin or imipramine did not display any significant locomotor impairment. Leptin, in a similar way that of the antidepressant imipramine, prevented the core characteristics of depressive-like behavior induced by LPS, showing its antidepressant-like properties in this model.

Furthermore, by using the number of entries in the center of the open field as a parameter of anxiety-like behavior, we observed that LPS-induced anxiety-like behavior was not prevented by leptin administration. This absence of anxiolytic effect by leptin in this inflammatory model of depression must be confirmed in future studies. In a previous study of our group, we did not observe anxiety-like alterations in the plus maze test 24 h post LPS, only 1.5 h after the endotoxin challenge, a time-point related to sickness behavior (22).

A number of studies advocate for abnormalities in dopaminergic neurotransmission in major depressive disorder (54). Initially, these data came from studies showing a reduced DA turnover (decreased homovanilic acid levels) in cerebrospinal fluid (CSF) of these patients (55, 56). Subsequently, neuroimaging studies showed an important impairment in L-DOPA uptake in the striatum of depressive individuals, as well as abnormalities in dopamine transporter (DAT) and dopamine receptors functioning (57, 58).

Some studies have reported that immune-inflammatory stimuli, such as LPS, or direct cytokine administration can severely affect dopaminergic system to mediate depressive symptoms, such as anhedonia and psychomotor retardation (59). In fact, it was described that bacterial endotoxin and pro-inflammatory cytokines (IL-1β, IL-6, and TNF-α) reduce ventral striatum responses to reward (60), and decrease dopamine availability and its metabolites levels in CSF and striatum (34, 61). Not only this, it was previously reported that pro-inflammatory cytokines: (i) decrease the expression of vesicular monoamine transporter 2 (VMAT2), responsible for packaging cytosolic dopamine into vesicles for release, in rat synaptosomes, (ii) consume tetrahydrobiopterin (BH4) cofactor, which is an essential step for dopamine biosynthesis mediated by tyrosine hydroxylase (TH) (62), and (iii) increase the expression or function of the dopamine transporter (DAT). Altogether these mechanisms lead to a decrease in synaptic dopamine content.

As aforementioned, neuroinflammatory alterations observed in depression and modeled by inflammatory models of depression can compromise dopamine neurotransmission in brain areas related to motivation, such as striatum, and to the integration of motivation and cognitive control for decision making, such as PFC (63). Noteworthy, mPFC has a causal importance in regulating behavioral despair in the forced swimming test since it was previously shown that different physiologically-defined mPFC neural populations can regulate despair-like states, one being selectively inhibited, and the other selectively activated (64). Since dopamine is the main neurotransmitter in the PFC as well as related to the regulation of motivated behavior in the striatum (65), we chose, in the present study, these two brain areas for evaluation.

Leptin seems to regulate brain-reward circuit mainly by altering dopamine signaling. Evidences for leptin regulation of reward circuit are the reversal of drug-seeking behavior induced by heroin withdrawal (66) and increases in amphetamine-induced locomotor sensitization (67). In this context, an elegant study demonstrated that a peripheral injection of leptin increased the expression and phosphorylation of TH in the NAcc as well as normalized deficits in dopamine release and its cytosolic contents in leptin-deficient-mice (ob/ob mice) (68). Additionally, a recent study showed that the ablation of the signal transducer and activator of transcription-3 (STAT3), a main transcriptional factor involved in leptin receptor (long-form) activation, importantly impaired axonal dopamine overflow in VTA and reduced the expression of TH and dopamine D1 receptors (69).

In the present study, we are showing the participation of D1-like and D2/D3 dopamine receptors in leptin antidepressant-like action in LPS-induced depressive-like behavior. Despite evidence indicating the involvement of dopaminergic neurotransmission in leptin effects, no previous study had evaluated the participation of dopamine receptors in the antidepressant mechanism of this hormone. Indeed, strong evidences advocates for the participation of dopamine receptors in depression: (i) chronic treatment with antidepressants of different classes increase the striatal expression of dopamine D2/D3 (70) and D1-D5 receptors (71), (ii) dopamine D1 receptor acute stimulation results in antidepressant-like effect in the forced swimming and learned helplessness tests, whereas its blockade hinders the effect of antidepressant drugs in these tests (35, 72), (iii) dopamine D2 receptors stimulation exerts antidepressant-like effects in the forced swimming test, while D2 receptors antagonism prevent the effects of classical monoaminergic agents (73–75), (iv) the anti-immobility effect of dopamine reuptake inhibitors in the forced swimming test depends on D1- and D2-like receptors activation (76), and (v) blockade of D2- and D1-like receptors reduced the sucrose consumption in two bottle preference test (77) and inhibited conditioned place preference induced by sweet solutions (sucrose, fructose, and glucose) (78, 79).

In our study, the blockade of D1- and D2-like dopamine receptors equally abrogated leptin effects in the forced swimming and sucrose preference tests. It is worth to mention that leptin regulatory effect in hedonic feeding is dependent on dopamine signaling through D2 receptor activation, and is inhibited by the pharmacological and genetic deletion of this receptor (40). Also, leptin-deficient mice presented reduced D2 receptor binding affinity in the striatum and leptin treatment restored this deficit (80). Therefore, our results corroborate previous evidence about the involvement of dopamine signaling in leptin central actions, by showing the participation of dopamine receptors in the antidepressant-like effects of this hormone.

Regarding IL-1β levels, our results point to a possible anti-inflammatory mechanism mediating leptin antidepressant-like effect. In this study, we assessed IL-1β levels as a parameter of active inflammation in relevant brain areas related to mood control. Indeed, leptin plays an important regulatory role in the immune system, in addition to its already established neuroendocrine functions. The levels of leptin seem to rapidly increase in acute inflammatory conditions, such as cholecystectomy and sepsis, particularly favored by cytokines such as TNF-α, IL-6, and IL-1β (81).

Nevertheless, it was also reported that leptin exhibits some peripheral anti-inflammatory properties (82). In the brain, the peripheral administration of leptin seem to regulate IL-1β transcripts in many regions, such as the hypothalamus, hippocampus, cortex, and cerebellum (83). Also, leptin increases IL-1β cleavage and release in sham (not immune stimulated) microglia (84). Furthermore, it was reported that leptin also increases IL-1β receptor antagonist expression in brain areas (85). It is important to highlight that IL-1β acts as a pleiotropic pro-inflammatory cytokine, inducing its own synthesis and of other pro-inflammatory cytokines, such as IL-18 and IL-6 (86).

According to our results, leptin plays an apparent anti-inflammatory effect as it reduced IL-1β levels in the PFC and striatum of mice challenged with LPS. It is important to note, that in our experimental conditions, we tested leptin effects in an established pro-inflammatory milieu induced by LPS, but not in control conditions, as performed in the abovementioned studies. Additionally, a recent study demonstrated leptin's anti-inflammatory actions in primary glial cell cultures challenged by the pro-inflammatory cytokines TNFα, IL-1β, and IFN-γ (87).

Our results also pointed to an interesting involvement of D1-like receptors in leptin anti-inflammatory effect in the PFC. Previous evidence showed that the activation of dopamine receptors can modulate cytokine production in immune cells (88). In this context, an elegant study showed that dopamine inhibits inflammasome signaling and IL-1β synthesis through activation of dopamine D1 receptors (89). Recently, another study confirmed these findings by demonstrating that a selective agonist of D1 receptors (A68930) exerts important anti-inflammatory effects in the brain of mice submitted to intracerebral hemorrhage. Of note, a D1 agonist partially inhibited the activation of microglia and decreased Nod-like receptor protein (NLRP) 3, caspase 1, and IL-1β expression (90). Therefore, our results on the involvement of D1-like receptors in leptin anti-inflammatory effects are in line with the previous findings of the involvement of these receptors in brain inflammatory alterations, and represent a potential mechanism involved in leptin antidepressant-like effect.

Neurotrophic factors are critical regulators of the formation and plasticity of neuronal networks. BDNF is an abundant neurotrophin in the brain involved in neuronal proliferation and differentiation (91). Experimental studies have demonstrated that stress reduces BDNF expression or hippocampal activity being this reduction prevented by the treatment with antidepressant drugs (92). Similar decreases in BDNF levels have been reported in the plasma of depressive patients, and the increase in this neurotrophin has been suggested as a predictor of antidepressant response (93).

In our experimental conditions, 24 h post LPS, we did not observe decreases in hippocampal BDNF levels, although pretreatment with both imipramine and leptin increased this parameter compared to saline and LPS-treated animals. This absence of alteration in hippocampal BDNF levels 24 h post LPS was also observed in previous studies of our research group (25).

This variability in BDNF 24 h post LPS, observed in our results, might be explained by the release of the immature form of BDNF, namely proBDNF, which has apoptotic effects. Regarding leptin effects, a previous study reported that the subcutaneous administration of this peptide increases BDNF levels in the hippocampus of non-obese mice and simultaneously induces an antidepressant-like effect in forced swimming test (94). Besides this, the intrahippocampal injection of the BDNF receptor blocker (K252a) fully abolished leptin antidepressant effect (94). Therefore, these findings corroborate our results of leptin ability to increase hippocampal BDNF levels and suggests its possible involvement in the mechanism of leptin antidepressant-like effect. Despite this, we found that the pharmacological blockade of dopamine receptors did not affect leptin effects in BDNF levels, which lead us to hypothesize that these receptors are not directly involved in this action of leptin. However, this proposition needs to be better explored.

The present study has some limitations. Indeed, human depression is a syndrome, comprising symptoms such as depressed mood, diminished interest or pleasure, fatigue or loss of energy, social withdrawal and cognitive dysfunction, among others, and for this reason should be evaluated in experimental models by behavioral tests related to these symptoms. In the present study, we focused on core alterations of depression, namely behavioral despair and anhedonia and in a less extent anxiety-like alterations. Hence, it is important to address, in future studies, leptin's effects in other behavioral tests related to anxiety, social interaction, and cognitive function. Furthermore, it is important to determine alterations in dopaminergic receptors in brain areas of mice exposed to LPS depression model, to better address the participation of these receptors in this inflammatory model as well as the effects of leptin administration in these receptors.

## Conclusion

In summary, the present study demonstrated leptin's ability to rescue LPS-induced depressive-like behaviors including anhedonia and despair-like behavior, and some immune changes, namely increases in IL-1β levels in the PFC and striatum. This is the first study showing the involvement of dopamine receptors in the antidepressant-like effects of leptin, suggesting the participation of D1-like receptors in this hormone immunoregulatory effects in the PFC. Of note, a previous study have pointed to hippocampus as the main site of leptin antidepressant effects (43). Therefore, considering the broad impact of depression and the marked limitations of the existing antidepressants, identifying the key brain regions and molecular targets that mediate leptin's antidepressant action may provide new insights into the pathogenesis of depression and the development of novel therapeutic strategies for the treatment of this illness.

## Author Contributions

RC conceived the study, conducted the experiments, and wrote the first draft. AF, VT, CM, AQ, and NG conducted the experiments, and analyzed and interpreted the data. MM analyzed the data, interpreted the data, and provided intellectual input to the manuscript. DM and AC conceived the study, supervised the experiments, analyzed and interpreted the data, and provided intellectual input to the manuscript. All authors have reviewed and approved the manuscript prior to submission.

### Conflict of Interest Statement

The authors declare that the research was conducted in the absence of any commercial or financial relationships that could be construed as a potential conflict of interest.
